# Perspectives from metabolomics in the early diagnosis and prognosis of gestational diabetes mellitus

**DOI:** 10.3389/fendo.2022.967191

**Published:** 2022-09-28

**Authors:** Muqiu Zhang, Huixia Yang

**Affiliations:** Department of Obstetrics and Gynecology, Peking University First Hospital, Beijing, China

**Keywords:** gestational diabetes mellitus (GDM), metabolomics, newborn infant, diagnosis and prediction, type 2 diabetes

## Abstract

Gestational diabetes mellitus (GDM) is one of the most common metabolic disorders in pregnant women. The early detection of GDM provides an opportunity for the effective treatment of hyperglycemia in pregnancy, thus decreasing the risk of adverse perinatal outcomes for mothers and newborns. Metabolomics, an emerging technique, offers a novel point of view in understanding the onset and development of diseases and has been repeatedly used in various gestational periods in recent studies of GDM. Moreover, metabolomics provides varied opportunities in the different diagnoses of GDM from prediabetes or predisposition to diabetes, the diagnosis of GDM at a gestational age several weeks earlier than that used in the traditional method, and the assessment of prognosis considering the physiologic subtypes of GDM and clinical indexes. Longitudinal metabolomics truly facilitates the dynamic monitoring of metabolic alterations over the course of pregnancy. Herein, we review recent advancements in metabolomics and summarize evidence from studies on the application of metabolomics in GDM, highlighting the aspects of the diagnosis and differential diagnoses of GDM in an early stage. We also discuss future study directions concerning the physiologic subtypes, prognosis, and limitations of metabolomics.

## Gestational diabetes mellitus

Gestational diabetes mellitus (GDM) is a common metabolic disorder that is defined as any degree of glucose intolerance with onset or first recognition during pregnancy (1). GDM affects approximately 14% of pregnancies worldwide, representing approximately 18 million births annually (2). Being overweight, being of advanced maternal age, having micronutrient deficiencies, and having a family history of insulin resistance and/or diabetes are risk factors for GDM (3). Meanwhile, the risk of GDM is increased in case of disturbances in the metabolism of the three nutrients, namely, carbohydrates, fat, and protein (4).

In clinical practice, the diagnosis of GDM is accompanied by several challenges. It is challenging to differentiate GDM from prediabetes or predisposition to diabetes in some cases; moreover, there is a possibility of heterogeneity of physiologic processes underlying hyperglycemia in women with GDM. Hyperglycemia in pregnancy is associated with adverse maternal and prenatal outcomes; however, there is a lack of international consensus regarding the timing of the screening method and optimal cutoff points for the diagnosis and intervention of GDM (5). Routine screening of the general population, including pregnant women, helps in identifying patients with prediabetes or predisposition to diabetes (5). Furthermore, based on the metabolic abnormality in insulin sensitivity or deficient insulin secretion, patients with GDM can be classified as cases with predominant insulin sensitivity defects, predominant insulin secretion defects, or normal glucose tolerance (6).

**Figure 1:**
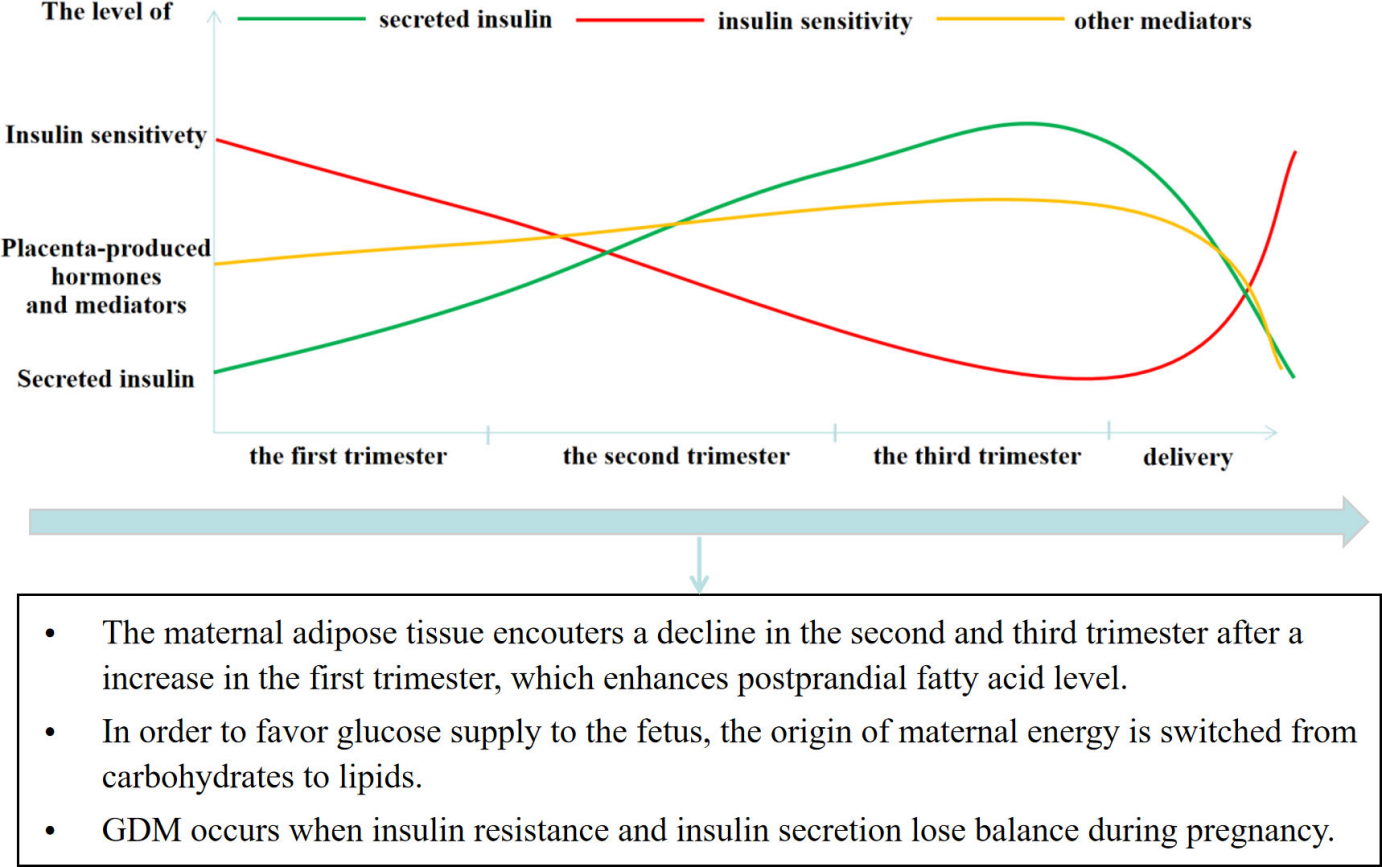
The glucose metabolism alteration and its influences in pregnancy.

The metabolism of a pregnant woman undergoes constant alterations once the pregnancy starts to support fetal development. Increased serum insulin secretion and insulin resistance are the most obvious maternal metabolic changes (**Figure 1**). During pregnancy, the amount of insulin secreted by pancreatic β cells steadily increases until the peak in the third trimester and returns to the normal level after delivery (7, 8). Along with increased insulin secretion, there is a decrease in maternal insulin sensitivity at the end of the first trimester, which continues until before delivery (9, 10). The insulin receptor signal is affected by increased placental lactogen, placenta-derived human growth hormone, progesterone, cortisol, prolactin, and other hormones, leading to GDM (11). Pathophysiologically, GDM occurs when there is an imbalance in insulin sensitivity and secretion during pregnancy. In detail, the level of insulin secreted by pancreatic β cells is unable to keep up with the increasing insulin resistance (12).

GDM develops among women with normal glucose before pregnancy in a more occult way throughout trimesters. Women with GDM are usually more likely to experience pregnancy-related complications, including high blood pressure and large birth weight (2), which are improved by effective glycemic control. Thus, timely detection and control of GDM are dispensable for the decrease in pregnancy-related complications (13, 14). Furthermore, children born to mothers with GDM are at high risk of suffering from type 2 diabetes mellitus and obesity at an early age (15–17). Therefore, it is necessary to put greater efforts into exploring GDM, particularly with respect to early diagnosis and prognosis.

There are alterations in metabolism during pregnancy, and hyperglycemia is a metabolic disorder. In this review, we discuss updates in metabolomics and summarize studies on the application of metabolomics in GDM, highlighting aspects of the diagnosis and differential diagnoses of GDM in an early stage. We also mention future study directions concerning the physiologic subtypes, prognosis, and limitations of metabolomics.

## Metabolomics in gestational diabetes mellitus studies

The composition of the metabolome, the complete set of metabolites and lipids in a biological system, directly reflects the physiological status, gene expression, and environmental stimuli of the biological system. Changes in the concentration or rate of transformation of metabolites under pathophysiological processes, such as aging and diseases, can be used as biomarkers for the diagnosis and prediction of clinical outcomes. Metabolomics has the advantage of recording disease-relevant metabolic changes and recognizing new biomarkers of disease processes (18). After further verification, these important metabolites can be used for disease diagnosis, therapeutic response assessment, or even predicting susceptibility to diseases (19).

Metabolomics has been successfully used to distinguish many disease-associated metabolite types in cancer, inflammatory bowel disease, asthma, diabetes, traumatic brain injury, metabolic syndrome, Parkinson’s disease, and so on (20–20). In a series of studies, the changes in metabolites were analyzed in the specific stages of pregnancy in women with pregnancy-associated complications, such as preeclampsia and GDM (20–22). The results of a metabolomics study interpreted the disease after the integration of multiple factors, including disease process, environmental exposures, demographic variations, and dietary habits, which are also the origin of study heterogeneity (23). Therefore, a successful metabolomics study calls for considerate preparations, which include consideration of confounding variables, powerful calculation for sample size, and standard sample extraction and storage (24). In metabolomics studies of GDM, the known confounding variables include ethnicity, maternal age, pregravid body mass index, family history of diabetes, history of GDM, and newborn sex (24). Furthermore, statistical power analysis should be performed to form an appropriate sample size (25). Metabolomics, as an important part of the biological system, mainly analyzes blood, urine, and feces and then studies the small-molecule metabolites of various metabolic pathway matrices and products (26). In the studies of GDM or other gestation-associated disorders, the serum in the umbilical cord and amniotic fluid is also collected for analysis. In rare cases, placenta or mothers’ hair is collected for analysis. In general, samples should be stored at −80°C for short‐term periods. Usually, complex and time-consuming sample preparation procedures are not used, except for the collection of samples of the placenta or mothers’ hair.

In the process of metabolomics, proton nuclear magnetic resonance (NMR) and mass spectrometry (MS) are effective tools for analyzing the molecular composition of a sample. Liquid chromatography (LC), gas chromatography (GC), and capillary electrophoresis (CE) are used for metabolite separation. LC, GC, or CE combined with MS or NMR spectroscopy are the most commonly used metabolomics platforms (27). Proton NMR is widely used in metabolomics studies due to its nondestructive nature and ability to simultaneously measure many organic compounds present in biological samples. However, the low sensitivity of proton NMR, which permits the detection of metabolites only at the micromolar level, is the major limitation of NMR as a comprehensive technique (28). Conversely, MS-based methods provide increased sensitivity and the ability to assay a diverse range of cellular metabolites over a varied polarity range. As such, in clinical metabolomics, NMR has a trend to be superseded by the evolved MS-based methods (23). Untargeted and targeted approaches are the two analytical strategies commonly used in metabolomics (29). The untargeted approach detects metabolites without an *a priori* hypothesis and is more suitable for studies focused on assessing potential biomarkers or metabolic mechanisms for diseases (29–32). The targeted approach analyzes the specific kind of metabolites and the relative metabolic pathways with *a priori* information and is used for biomarker validation and studying a specific biological pathway (29–32). Data generated by untargeted approaches are extremely complex, and the majority of peaks in the profile are not identifiable. Furthermore, the concept of fingerprinting in an untargeted approach was initially developed for microbiology to classify microbial species but is not useful in clinical applications (33). Currently, metabolomics datasets for annotating the spectral features from the untagged approach are not available (34). Conversely, in clinical applications, data processing and normalization are critical in untargeted metabolomics studies. Profile clustering may be used for the diagnosis of patients. Targeted metabolomics is an important workflow because of the higher sensitivity and selectivity and the validation and expansion of results from the untargeted analysis (35).

In addition to high efficiency, ease of interpretation, and acceptable cost, clinical practice poses an additional requirement for metabolomics, and superior reproducibility, particularly in the case of disease prediction. However, metabolomics usually generates a long list of metabolites, which could not be directly used in clinical practice. Advanced algorithms are needed to define and integrate metabolites with the utmost potential. Traditionally, univariate analysis and logistic regression are performed. Recently, machine learning, a data analysis technique that develops algorithms for predicting outcomes by “learning” from data, has been increasingly highlighted as a competitive alternative to regression analysis. Machine learning has been mainly classified into supervised and unsupervised. Hierarchical clustering, principal component analysis, and self-organizing maps are the unsupervised methods that have been used in analyzing metabolomics data. Supervised methods include support vector machines, partial least squares, analysis of variance, k-nearest neighbors, and discriminant function analysis (36). Machine learning outperforms conventional regression in terms of its ability to capture nonlinearities and complex interactions among multiple predictive variables (37). A few studies on the prediction of GDM have been conducted for comparing the performances of machine learning and logistic regression. Liu et al. (38) developed a machine learning-based prediction model for GDM in women within early pregnancy and compared it with a traditional logistic model. The machine learning method with extreme gradient boosting had similar performance in validation but was better in calibration. Meanwhile, the results of studies comparing machine learning with logistic regression should be critically interpreted. Ye et al. (39) and Wu et al. (40) used a series of machine learning methods to select candidate predictors and build predictive models for GDM. As a result, not all methods in machine learning outperformed logistic regression. For instance, in a study by Ye et al. (39), only three out of eight machine learning methods (AdaBoost, Vote, and LGB) invariably outperformed logistic regression in both external validation and calibration. In a study by Wu et al. (40), the machine learning algorithms had an inferior balance for sensitivity and specificity (Youden index) than the traditional logistic regressions, except for deep neural networks. Furthermore, the models from machine learning algorithms were inclined to have high specificity but low sensitivity (40). Meanwhile, the sample size and the number of variables are another concern when using machine learning.

## Comparison of metabolic profiles between gestational diabetes mellitus and type 2 diabetes mellitus by metabolomics

The diagnostic paradigm of GDM is a problem across different guidelines throughout the world. The American Diabetes Association (ADA) formally classifies GDM as “diabetes first diagnosed in the second or third trimester of pregnancy that is not overt (preexisting type 1 or type 2) diabetes” (13). GDM is typically diagnosed using an oral glucose tolerance test between 24 and 28 weeks of gestation. However, the International Association of Diabetes and Pregnancy Study Group also recommends screening for overt diabetes at the first antenatal visit. The ADA standard might have difficulties in distinguishing patients with true GDM from those patients with either prediabetes or predisposition to diabetes. There is a lack of international consensus on the screening and diagnosis of GDM; furthermore, the intentions of early diagnosis of GDM and differentiation from prediabetes or predisposition to diabetes have not been obtained yet. Therefore, metabolomics is rendered to have great expectations in the discernment of GDM.

Several investigations of metabolite profiles have facilitated the identification of potential mechanistic pathways for both diabetes and GDM, thus helping detect their similarities and disparities. Protein metabolism reflected by changes in plasma amino acid concentrations is reported with high frequencies (41). Branched-chain amino acids (BCAAs), including valine, leucine, and isoleucine, are repeatedly reported to be associated with risk factors for diabetes (42). In contrast, elevated levels of BCAAs in women with GDM compared with controls have not been observed in all circumstances. The pioneering study by Metzger et al. (43) observed elevated levels of BCAAs in women with GDM at 30–39 weeks of gestation, which was also later confirmed by Butte et al. (44). In another study, fasting maternal plasma carnitine (total, free, and acyl-carnitine), beta-hydroxybutyrate, free fatty acids, glycosylated hemoglobin, and 21 amino acids were assayed at 30–33 weeks of gestation. Of the 21 amino acids, only methionine, glycine, alanine, citrulline, and ornithine levels were found to be significantly higher in the study group than those in the control group. Meanwhile, Pappa et al. (45) delineated that in GDM, ketogenic amino acids and the branched-chain amino acid isoleucine are released at low rates from the skeletal muscles and mostly catabolized in the liver rather than in the peripheral tissues. Along with BCAAs, alterations in the metabolic by-products of protein, including aromatic amino acids, sulfur-containing amino acids, and asymmetric dimethylarginine, contribute to the development of diabetes and insulin resistance (46). However, inconsistent results have been drawn in various studies on GDM (46, 47). Further study in larger populations is required for explaining the interactions between GDM and the metabolism of proteins. The major components of triacylglycerols, non-esterified fatty acids (NEFAs), are the energy source for many body tissues. Increased circulating levels of NEFAs have been well described in studies on insulin resistance and type 2 diabetes (48, 49). Similarly, upregulated levels of NEFAs in women with GDM were detected in the third trimester of pregnancy, which might be aggravated by an increase in dietary intake of polyunsaturated and saturated fatty acids during pregnancy (50, 51). However, few metabolomics studies are conducting head-to-head comparisons between GDM and diabetes. Moreover, it is important to match the confounding factors including gestational time, techniques used in metabolomics, and other metabolic disorders when applying metabolomics in patients with GDM and diabetes.

## Early diagnosis of gestational diabetes mellitus by metabolomics

According to Clarke et al. (52), the early diagnosis of GDM and timely treatment at an average of 17 weeks of gestation minimized neonatal adverse events. However, the traditional methods based on the oral glucose tolerance test often detect GDM at 24–28 weeks of gestation, thus leaving patients with GDM untreated for weeks and causing deleterious effects on the fetus. Hence, there is a need for examining novel diagnostic biomarkers for GDM to facilitate early detection and treatment.

According to metabolomics, abnormal metabolism occurs before the GDM attack (53). Generally, GDM is a multifaceted condition that involves changes in various metabolic pathways including amino acids, carbohydrates, lipids, and purines (47). A series of studies have attempted to determine biomarkers in urine, amniotic fluid, or plasma for diagnosing GDM at 14–25 weeks of gestation. Pinto et al. (54) performed NMR spectroscopy to identify alterations in metabolites in maternal plasma and lipids extracted at 2–21 weeks of gestation. Compared with those who did not develop GDM, the potential patients with GDM had increases in plasma valine and pyruvate, with decreases in proline, urea, and 1,5-anhydroglucitol. In the study by Hou et al. (55), liquid chromatography-mass spectrometry (LC-MS), GC, and NMR were performed on maternal serum from pregnant women with GDM and normal glucose tolerance. The results showed that the changes in free fatty acids, BCAAs, lipids, and organooxygen compounds differentiated the GDM groups from the healthy group. Furthermore, Hou et al. (55) built models for the risk prediction of GDM based on data from metabolomics and key clinical parameters. In addition, increases in acetate, creatine, creatinine, choline, 3-hydroxyisovalerate, and hydroxyisobutyrate and decreases in trimethylamine N-oxide and betaine in the first trimester are also considered potential signs of developing GDM (56, 57).

Zhu et al. (58) explored metabolomics markers and developed a panel for the early diagnosis of GDM, which paved the way for clinical practice. Time-of-flight GC-MS was performed in cohorts from three population-based studies conducted by different centers, which included 168 patients with GDM and 622 normal controls. The general study cohort had uniform diagnostic criteria but heterogeneity in ethnicity. Ten-fold cross-validated Lasso regression was used to identify predictive metabolomics markers at 10–13 and 16–19 weeks of gestation for GDM. Purinone metabolites at both 10–13 and 16–19 weeks of gestation and amino acids, amino alcohols, hexoses, indoles, and pyrimidine metabolites at 16–19 weeks of gestation were positively associated with GDM risk. Finally, Zhu et al. (58) found that a 17-metabolite panel at 10–13 weeks of gestation and a 13-metabolite panel at 17–19 weeks of gestation outperformed the model using conventional risk factors, including fasting glycemia.

## Longitudinal metabolomics in studies on gestational diabetes mellitus

The drawback of most of the published studies is measuring maternal metabolic profiles at only one time point during pregnancy or pooling metabolome data across trimesters. Metabolite alterations may occur in conjunction with substantial metabolic changes in the maternal body during different trimesters of pregnancy, highlighting the value of longitudinal metabolomics research at different pregnancy stages. Several studies have also been dedicated to determining the dynamic alterations in metabolites across different time points during pregnancy, which completely marked the metabolic profiles of GDM.

The pioneering study of GDM by longitudinal metabolomics was conducted by Law et al. (59). LC-MS untargeted metabolomics for maternal plasma was performed along with innovative sample preparation and multilevel statistical methods. All participants were scheduled for three antenatal visits at 11–14, 23–27, and 29–33 weeks of gestation. Compared with the healthy controls, the participants who developed GDM showed a reduction in polyunsaturated phospholipids in the first trimester, independent of the stage of gestation and steroid hormones. In, 2017, Law et al. (60) conducted another longitudinal metabolomics study on GDM. In this follow-up study, urine samples were collected at every antenatal visit during the three trimesters. LC-MS untargeted metabolomics was performed to assess the differences in the urinary metabolome of patients with GDM and healthy controls over the course of pregnancy. Accordingly, before placental hormones or the fetoplacental unit could have produced any physiological effect, the tryptophan–kynurenine pathway was activated in patients with GDM, ultimately leading to uric acid production. The results of Law et al. (60) supported the notion that GDM is a predisposed condition and can be predicted by urinary metabolome countering tryptophan and purine. The two studies by Law et al. (59, 60) set an important role of longitudinal metabolomics in the early diagnosis and prediction of GDM.

Zhao et al. (61) performed MS-based untargeted metabolomics in pregnant women with GDM and healthy controls in their first and second trimesters to investigate the trimester-specific alterations of metabolites related to GDM. In the first trimester, the GDM group had 31 significantly altered metabolites, which were mainly attributed to purine metabolism, fatty acid β-oxidation, and urea cycle and tricarboxylic acid cycle pathways. In the second trimester, significant changes in fold changes across trimesters were detected in six amino acids, lysophosphatidylcholine, and uric acid, which might have contributed to the occurrence and progression of GDM (61). The study by Zhao et al. (61) truly recognized the dynamic monitoring of metabolic alterations by metabolomics over the course of pregnancy.

Apart from GDM, obesity and hypertensive disorders are also common metabolic disorders in pregnancy. It has been suggested that the so-called metabolic disturbances caused by GDM are confused with other concurrent metabolic disorders. Kivelä et al. (62) explored the metabolic profiles of pregnant women suffering from all three metabolic complications. Proton NMR was performed on blood samples collected at a median of 13, 20, and 28 weeks of gestation. Across all three time points, women with obesity had significantly higher levels of very-low-density lipoprotein, fatty, and amino acids and more adverse metabolic profiles. Meanwhile, many of the adverse metabolic profiles associated with GDM were rendered nonsignificant after adjustment for body mass index (62).

## Metabolic profiles of women with gestational diabetes mellitus in different physiologic subtypes

For women who are not pregnant, hyperglycemia results from a defect in either insulin secretion or insulin sensitivity (63), which supports the possibility of the physiologic heterogeneity of GDM. According to the metabolic abnormality in insulin sensitivity or deficient insulin secretion, GDM can be classified into three physiologic subtypes: insulin sensitivity defects, insulin secretion defects, and normal glucose tolerance (6). It is of clinical importance to classify GDM into physiologic subtypes, which are associated with risks of adverse perinatal outcomes (64). For instance, women with GDM with high insulin resistance have higher rates of preterm delivery, labor induction, Cesarean section, neonatal hypoglycemia, and neonatal intensive care unit admissions (64, 65). Several lines of evidence indicate the different metabolic profiles existing in patients with GDM with the three physiologic subtypes. Obesity-related factors, including pre-pregnancy overweight and elevated gestational weight gain in the first trimester, are specific to the insulin-resistance subtype (66). Layton et al. (67) measured lipid markers in fasting plasma collected during the second trimester for characterizing lipid profiles in women with different physiologic subtypes of GDM. Women with GDM characterized by a predominant insulin sensitivity defect had significantly higher triglycerides, lower high-density lipoprotein, and higher NEFA than those with GDM and normal glucose tolerance. Women with GDM characterized by a predominant insulin secretion defect had higher NEFA levels than those with GDM and normal glucose tolerance. Currently, no study has been conducted to determine the metabolic characteristics of GDM with different physiologic subtypes by metabolomics. The physiologic subtypes of GDM are closely associated with the prognosis of mothers and newborns; thus, it is essential to perform studies on the physiologic subtypes along with data of metabolomics and clinical indexes, which help detect indications of abnormal metabolism belonging to different subtypes of GDM.

## Limitations of metabolomics in the clinical practice of gestational diabetes mellitus

There are some limitations of metabolomics in the clinical practice of GDM. First, the process of metabolomics needs higher efficiency. The early detection and control of GDM result in less adverse perinatal outcomes. It usually takes weeks to months before clinicians obtain the final outcomes of metabolomics. According to the ADA standard, the disparity between the oral glucose tolerance test and metabolomics is approximately 10 weeks. It is essential to enhance the efficiency in the process of metabolomics. Second, obvious heterogeneity and low reproducibility exist in the present studies of GDM concerning metabolomics, which is a complication for clinicians in setting a definite cutoff value for one type of metabolite. The differences in GDM diagnostic criteria used, variation in analytical platforms used, analysis of different types of specimens, and disparity in the inherent characteristics of the cohort population are the main sources of heterogeneity (62). Therefore, future multicenter metabolomics studies on GDM are proposed using unified diagnostic criteria, longitudinal supervision of metabolites, and efficient data processing methods to cater to clinical practice.

## Conclusion

Metabolomics, an emerging technique, offers a new point of view in understanding the onset and development of diseases. In recent studies of GDM, metabolomics has been repeatedly used in various gestational periods. Metabolomics is rendered to have great expectations in the different and early diagnoses of GDM. Longitudinal metabolomics truly facilitates the dynamic monitoring of metabolic alterations over the course of pregnancy. Furthermore, patients with GDM with different physiologic subtypes have different prognoses and metabolic backgrounds. It would be of clinical importance to perform metabolomics in consideration of physiologic subtypes of GDM and clinical indexes. In conclusion, metabolomics requires further improvement in terms of efficiency and uniform standards in practice.

## Author contributions

MZ and HY wrote the manuscript. All authors contributed to the article and approved the submitted version.

## Funding

This work was supported by the National Key Research and Development Program of China (No., 2021YFC2700700) and the National Natural Science Foundation of China (81830044).

## Conflict of interest

The authors declare that the research was conducted in the absence of any commercial or financial relationships that could be construed as a potential conflict of interest.

## Publisher’s note

All claims expressed in this article are solely those of the authors and do not necessarily represent those of their affiliated organizations, or those of the publisher, the editors and the reviewers. Any product that may be evaluated in this article, or claim that may be made by its manufacturer, is not guaranteed or endorsed by the publisher.
